# Effects of insulin-loaded chitosan-alginate nanoparticles on RAGE expression and oxidative stress status in the kidney tissue of rats with type 1 diabetes

**DOI:** 10.22038/IJBMS.2018.28463.6899

**Published:** 2018-10

**Authors:** Shirin Heidarisasan, Nasrin Ziamajidi, Jamshid Karimi, Roghayeh Abbasalipourkabir

**Affiliations:** 1 Department of Biochemistry, School of Medicine, Hamadan University of Medical Sciences, Hamadan, Iran

**Keywords:** Advanced glycation end, Antioxidant, Diabetes mellitus, Insulin, Nanoparticles, Oxidant

## Abstract

**Objective(s):**

Chronic hyperglycemia leads to activation of the advanced glycation end products (AGE)-receptor (RAGE) for AGE axis and oxidative stress, which promote diabetic renal damage. This study examines the effect of insulin-loaded trimethyl chitosan nanoparticles on the kidney tissue of diabetic rats.

**Materials and Methods:**

Twenty-five male Wistar rats were randomly divided into 5 groups: normal control (C), diabetic group without treatment (DM), diabetic group treated with chitosan-based nanoparticle (DM+NP, 1 ml by gavage), diabetic group treated with 8 IU/kg insulin-loaded trimethyl chitosan nanoparticles (DM+N.in, 1 ml by gavage), and diabetic group treated with 8 IU/kg trade insulin (DM+SC.in, 0.2 ml by subcutaneous injection). The animals were treated from weeks 8 to 10. At the end of the study, serum urea, creatinine, and uric acid were measured. Also, the level of AGE and RAGE mRNA expression, and oxidative stress markers were studied in the kidney tissue.

**Results:**

Insulin-loaded nanoparticles similar to trade insulin could significantly reduce urea, creatinine, and uric acid parameters, while the elevated total antioxidant capacity (TAC), thiol groups, and catalase activity also reduced total oxidant status (TOS) and malondialdehyde (MDA) levels (*P*<0.05). However, the reduction in AGE and RAGE mRNA expression is not statistically significant in both treatments. Of course, the influence of insulin-loaded trimethyl chitosan nanoparticles on the amelioration of all these parameters is higher compared to that of the injected form. No markedly significant differences were observed between these two kinds of treatments.

**Conclusion:**

This data reveals that insulin-loaded trimethyl chitosan nanoparticle is a better therapeutic approach than injected insulin.

## Introduction

Diabetic nephropathy (DN)-one of the important microvascular complications of diabetes-is an important cause of end-stage renal disease (ESRD) (1). Several mechanisms have been considered to be involved in the pathogenesis of DN and other diabetes-associated complications, such as the accumulation of advanced glycation end-products (AGEs) and oxidative stress (2). AGE that is formed as a result of the non-enzymatic glycation of protein, lipid, and nucleic acid molecules is an important pathogenic factor in the onset and progression of diabetes complications (3). Increased formation and decreased clearance are both responsible for accumulation of AGE in patients with DN. AGE accumulation in the kidney is associated with progressive changes in the kidney structure, changes of infiltration patterns, and ultimately the loss of glomerular function (4). Additionally, the AGE, through interaction with a cell surface receptor called RAGE, activates cell-signaling pathways that are involved in the control of processes such as apoptosis, proliferation, cell migration, inflammation, and autophagy (5). Oxidative stress also plays an important role in the pathogenesis of DN (2). Hyperglycemia not only results in the formation of reactive oxygen species (ROSs) such as superoxide and hydrogen peroxide, but also weakens antioxidative systems through the glycation of scavenging enzymes such as superoxide dismutase and catalase (6). Also, the AGE-RAGE interaction leads to the formation of ROS (7). Since hyperglycemia directly affects the microvascular complications of diabetes-especially in the kidney-glycemic control is the main goal of the treatment (8). Subcutaneous injection of insulin is widely accepted in diabetic patients, but it is invasive and thus has many disadvantages such as infections, hypoglycemia, peripheral hyperinsulinemia, and poor pharmacodynamic (9). Recent studies on oral drug delivery are based on increased bioavailability and higher absorption of oral peptide drugs (10). Orally administered insulin is non-invasive; it first reaches the liver through portal circulation, similar to the physiological pathway of insulin secretion (11). However, enzymatic degradation in the gastrointestinal tract, low stability of gastric pH, and the physical barrier of the intestinal epithelium decrease the bioavailability of oral insulin (12). But over the past decade, nanoparticles have been considered as a suitable vehicle for the oral delivery of insulin (11). Recently, natural or synthetic polymers have been used in the synthesis of carriers suitable for the oral administration of insulin. Chitosan-a natural cationic polysaccharide-has been extensively studied for the delivery of insulin because of its characteristics such as biocompatibility, biodegradability, non-immunogenicity, and non-toxicity. The ionic interactions between the cationic amine groups of chitosan and the anionic groups at the epithelial cell surface results in mucosal adhesion. It is also possible to temporarily open the tight junction of the intestinal epithelium and increase the permeability of this delivered protein (9). Alginate (Alg) is a natural anionic polysaccharide containing varying amounts of (1–4)-linked b-D-mannuronic acid (M) and a-L-guluronic acid (G), which are widely used in the bioencapsulation of drugs, proteins, and cells. The gelling properties of the guluronic residues with divalent ions such as calcium permit the formation of Alg matrices for gels, films, beads, pellets, microparticles, and nanoparticles (13). These pH-sensitive polymers are stable in the low pH of the stomach but dissolves in the alkaline pH of the intestine. This unique behavior of alginate protects insulin molecules along the path from mouth to intestine (14). The objective of the present study is to compare the ability of conventional insulin and oral insulin-loaded trimethyl chitosan nanoparticles in reducing blood glucose in streptozotocin (STZ)-diabetic rats. Furthermore, we examined the effects of insulin-loaded trimethyl chitosan nanoparticles on the AGE/RAGE axis and oxidative stress in ameliorating renal complications associated with type1 diabetes.

## Material and Methods

***Nanoparticle preparation***

Insulin-loaded trimethyl chitosan nanoparticle was a contribution from Dr. Kalantarian. Its characteristics are shown in Table 1. 

***Experimental design***

This study was performed at Hamadan University of Medical Sciences in 2016. Male Wistar rats (n= 25) weighing an average of 200 g (ranging from 8 to 10 weeks old) were housed in standard cages under controlled conditions of temperature (25±2 °C), lighting (12 hr light/dark cycles), and access to commercial rat chow diet and tap water *ad* li*bitum *throughout the acclimatization and experimental periods. All procedures for the treatment of animals were approved by the Research Committee of Hamadan University of Medical Sciences, Iran (Res: IR.UMSHA.REC.1395.112).

Diabetes mellitus type 1(DM) was induced by an intraperitoneal (IP) injection of STZ, (Sigma) (60 mg/kg; dissolved in 0.1 M sodium citrate buffer pH=4.5). DM was verified 72 hr later by measuring blood glucose level (after an overnight fast) using glucose oxidase reagent strips (Accuchek; Roche, Germany). Animals with a fasting blood sugar of ≥200 mg/dl were considered to be diabetic. 

**Table 1:** Physical characteristics of insulin-loaded trimethyl chitosan nanoparticles

Particle size (nm)	Particle size distribution	Zeta Potential (mv)	Drug Loading %	Entrapment Efficiency %
533	0.533	+20	48.83	97.67

**Table 2:** Body weight and serum biochemical parameters of rats (n = 5)

Parameters/Groups	C	DM	DM+NP	DM+N.in	DM+SC.in
Body weight (gr)	307.60±17.39	177.60±28.44^a^†	164.60±09.03	217.00±08.16 b†	239.40±10.24^b^†
Fasting blood sugar (mg/dl)	084.60±007.45	507.50±129.26^a^#	488.40±065.19	294.10±51.54 b#	268.30±75.51^b^#
Urea (mg/dl)	64.02±9.96	132.75±17.76 a#	132.41±17.86	92.5±15.77 b*	95.5±13.72 b*
Creatinine (mg/dl)	0.69±0.13	1.43±0.08 a#	1.38±0.17	1.09±0.13 b*	1.12±0.11 b*
Uric acid (mg/dl)	1.71±0.20	3.94±0.49 a#	3.91±0.71	1.91±0.43 b*	1.97±0.35 b*

Data are shown as mean ± SD.

C=Normal control, DM=Diabetic control, DM+NP=Diabetic group treated with nanoparticle, DM+N.in=Diabetic group treated with oral nanoinsulin , DM+SC.in=Diabetic group treated with subcutaneous injected insulin. Eight weeks after the induction of diabetes, treatment was carried out for two weeks.

a versus group C,

b versus group DM.

* *P*<0.05,

† *P*<0.01,

# *P*<0.001

Control rats were injected with only citrate buffer.The rats with DM were randomly divided into four groups. Eight weeks after the induction of diabetes, treatment was carried out for two weeks. The studied groups (n=5 in each group) were as follows: control (C), diabetic group without treatment (DM), diabetic group treated with chitosan-based nanoparticle (DM+NP, 1 ml by gavage), diabetic group treated with 8IU/kg insulin-loaded trimethyl chitosan nanoparticles (DM+N.in, 1ml by gavage), and diabetic group treated with 8 IU/kg trade insulin (DM+SC.in, 0.2 ml by subcutaneous injection).

Weight and blood glucose levels of rats were determined before STZ injection and three days, eight weeks, and 10 weeks after the induction of diabetes. All animals were sacrificed at the end of the 10^th^ week. Blood was collected by puncturing the inferior vena cava at the time of sacrifice. Sera samples were separated by 10 min of centrifugation at 1500g and were stored at −20 ^°^C until used for analysis. 

The kidney samples were dissected, washed rapidly with cold phosphate-buffered saline (PBS), frozen in liquid nitrogen, and stored at −70 ^°^C. 

***Serum biochemical parameters*** ***assay***

Serum urea, creatinine, and uric acid were assayed using proper kits (Pars Azmun, Iran)

***Preparation of kidney tissue homogenate***

Kidney samples were excised, rinsed with ice-cold PBS, and homogenized with liquid nitrogen. The homogenate was resuspended in ice-cold lysis buffer (10 mM (4-(2-hydroxyethyl)-1-piperazineethanesulfonic acid), 10 mM KCl, 1.5 mM MgCl_2_, 1 mM EDTA, 0.1% Triton X100, and protease inhibitor cocktail, pH=7.9) and incubated on ice for 20 min. This homogenate was centrifuged at 10,000 g for 15 min at 4 ^°^C (refrigerated centrifuge) and the supernatant was used for the experiments (15). The protein content of homogenate was measured by Bradford assay using crystalline bovine serum albumin (BSA) as standard (16).

***Total antioxidant capacity assay***

Total antioxidant capacity (TAC) was determined using ferric-reducing antioxidant potential (FRAP) assay. At low pH, the reduction of ferric tripyridyltriazine (F III -TPTZ) complex to the ferrous form, which has an intense blue color, can be monitored by measuring the change in absorption at 593 nm. This change is directly related to the combined or “total” reducing power of the electron-donating antioxidants present in the reaction mixture. An aqueous solution of known Fe(II) concentration (FeSO_4_.7H_2_O) is used as a standard (17).

***Total oxidant status assay***

The measurement of total oxidant status (TOS) is based on the oxidation of ferrous ion to ferric ion in the presence of oxidant agents in an acid solution (FOX1 assay). Detection is based on the formation of a colored complex between the generated ferric ion and xylenol orange with a maximal absorption at 560 nm. Hydrogen peroxide is used for the calibration of this assay (18).

***Lipid peroxidation assay***

A typical determination of aldehydes produced during lipid peroxidation in kidney tissue homogenate is based on reaction with thiobarbituric acid (TBA). The fluorescence intensity of its product is measured at 515nm excitation and 553nm emission wavelength. An aqueous solution of tetraethoxy propane is used as a standard.

***Thiol assay***

The spectrophotometric assay is based on the method of Ellman, in which 2,2-dithiobisnitrobenzoic acid (DTNB or Ellman’s reagent) is reduced by SH groups to form a 2-nitro-5-mercaptobenzoic acid anion. This anion has an intense yellow color with a maximal absorption at 412 nm and can be used to measure SH group contents (19).

***Catalase activity assay***

This method is based on the reaction of the enzyme sample with hydrogen peroxide as a substrate. The reaction stops with ammonium molybdate. The absorbance of the yellow complex of molybdate and hydrogen peroxide is measured at 374 nm (20).

***AGE assay***

Spectrofluorimetric detection according to the Kalousová method is used for the determination of fluorescent AGEs (21). Kidney homogenate is diluted 1:10 with PBS (pH=7.4) and fluorescence intensity was measured at 350 nm excitation wavelength and 440 nm emission wavelength. Fluorescence intensity was also expressed in arbitrary units (AU/mg pr).

***Real-time qPCR analyses for RAGE***

Total RNA was extracted from kidney tissue using RNX-Plus reagent (CinnaGen, Iran), according to the manufacturer’s protocol. Then, 1 µg of total RNA was reverse-transcribed into single strand cDNA using RevertAid™ First Strand cDNA synthesis kit (Thermo Scientific, Lithuania). Subsequently, quantitative real-time PCR was performed with Real Q Plus 2x Master Mix Green (AMPLICON, Denmark) on a LightCycler® 96 System (Roche Life Science, Deutschland GmbH Sandhofer, Germany). The amplification protocol comprised of one cycle at 95 ^°^C for 15 min followed by 40 cycles at 95 ^°^C for 20 sec, 58 °C for 30 sec, and then 72 °C for 30 sec. The gene to be studied was RAGE, while the internal control was a β-actin gene (BAC). Primer sequences were as follows: 

RAGE; Forward: 5′-GAGTCCGAGTCTACCAGATTCC-3′ 

Reverse: 5′-GGTCTCCTCCTTCACAACTGTC-3′.

β-actin; Forward: 5′ATCAGCAAGCAGGAGTACGAT-3′;

Reverse: 5′AAAGGGTGTAAAACGCAGCTC-3′. 

The 2^-ΔΔct^ method was used to evaluate the relative gene expression in the control and treatment groups.

***Statistical analysis***

The results were expressed as Mean ± SD. The inter-group variation was measured by one-way ANOVA followed by Tukey’s *post hoc* test. Statistical analysis was performed using the SPSS statistical software version 16.0. In all the analyses, *P*<0.05 was considered statistically significant.

## Results

***Effect of*** ***insulin-loaded trimethyl chitosan*** ***nanoparticles on body weight and serum biochemical parameters***

As shown in Table 2, diabetic rats exhibited decreased body weight compared to normal control rats (*P*<0.05) 10 weeks after STZ injection. 

After two weeks of treatment, oral insulin-loaded trimethyl chitosan nanoparticles similar to injected insulin could increase body weight significantly in comparison with untreated diabetic rats (*P*<0.01). However, there was no significant difference between these two treatment approaches. Compared to the normal control group, the diabetic group had higher (*P*<0.001) fast blood sugar (FBS) levels throughout the treatment process. 

**Figure 1:**
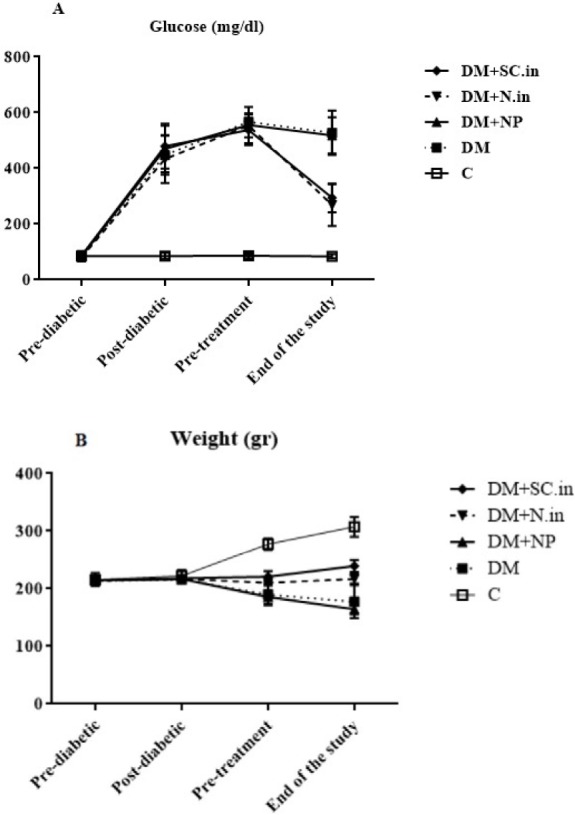
1A shows glucose level and 1B shows weight changes during 10 weeks in all groups

C=Normal control, DM=Diabetic control, DM+NP=Diabetic group treated with nanoparticle, DM+N.in=Diabetic group treated with oral nanoinsulin, DM+SC.in=Diabetic group treated with subcutaneous injected insulin.

**Figure 2:**
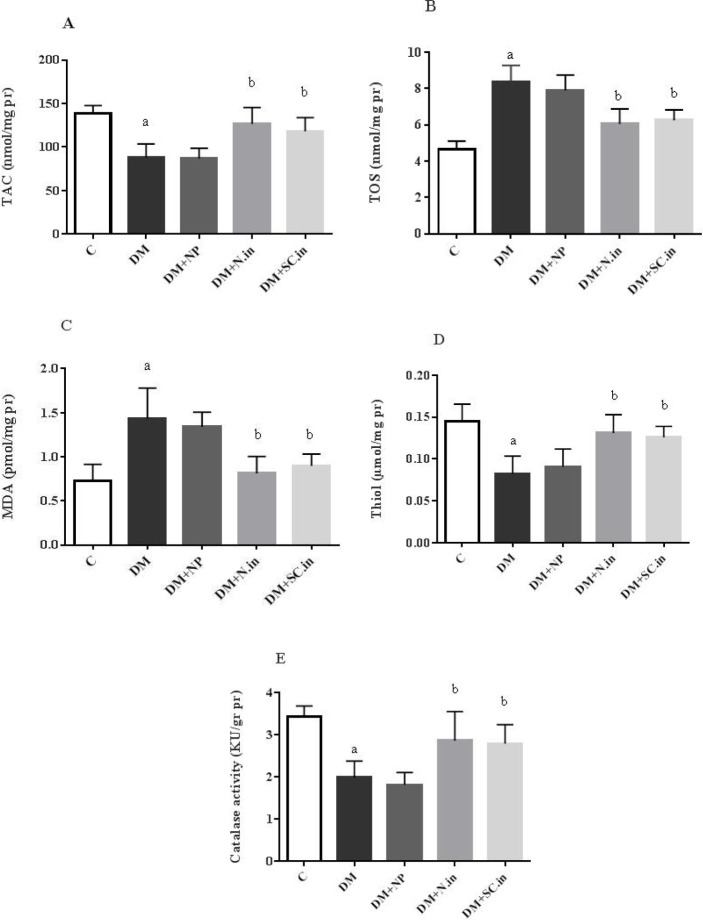
Effect of insulin-loaded trimethyl chitosan nanoparticle on oxidative stress parameters in kidney tissue (A-E) (n=5). Data are shown as mean ± SD. a; *P*<0.001 versus group C, b; *P*<0.05 versus group DM

C=Normal control, DM=Diabetic control, DM+NP=Diabetic group treated with nanoparticle, DM+N.in=Diabetic group treated with insulin-loaded trimethyl chitosan nanoparticle, DM+SC.in=Diabetic group treated with subcutaneous injected insulin.

**Figure 3:**
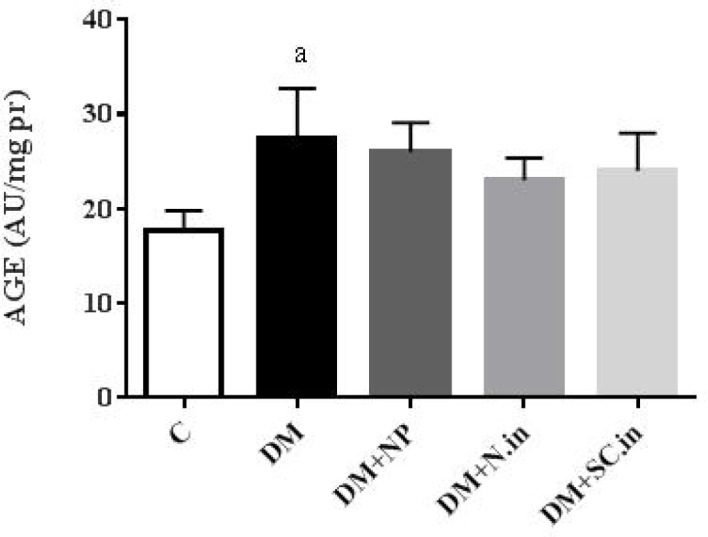
Effect of insulin-loaded trimethyl chitosan nanoparticle on advanced glycation end products (AGE) level in kidney tissue (n=5). Data are shown as mean ± SD. a; *P*<0.05 versus group C

C=Normal control, DM=Diabetic control, DM+NP=Diabetic group treated with nanoparticle, DM+N.in=Diabetic group treated with insulin-loaded trimethyl chitosan nanoparticle, DM+SC.in=Diabetic group treated with subcutaneous injected insulin.

**Figure 4:**
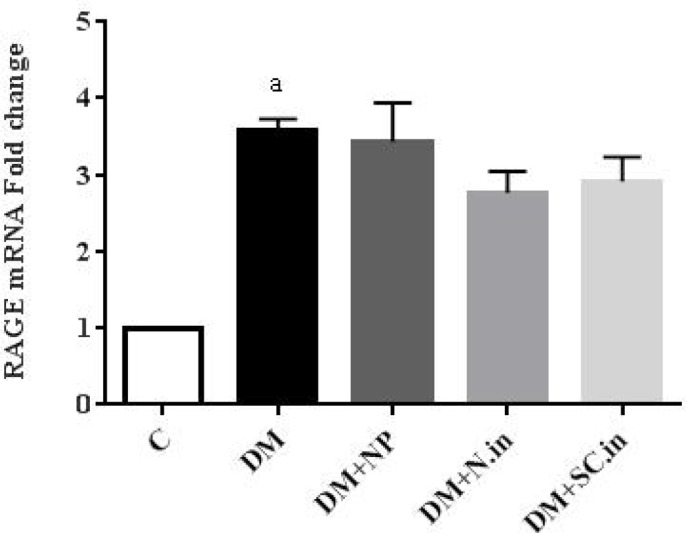
Effect of insulin-loaded trimethyl chitosan nanoparticle on advanced glycation end products receptor (RAGE) mRNA expression in kidney tissue (n=5). Data are shown as mean ± SD. a; *P*<0.01 versus group C

C=Normal control, DM=Diabetic control, DM+NP=Diabetic group treated with nanoparticle, DM+N.in=Diabetic group treated with insulin-loaded trimethyl chitosan nanoparticle, DM+SC.in=Diabetic group treated with subcutaneous injected insulin.

However, administration of oral insulin-loaded trimethyl chitosan nanoparticles similar to injected insulin could decrease FBS levels significantly in comparison with the untreated diabetic group (*P*<0.001).

However, insulin-loaded trimethyl chitosan nanoparticles had more potential in the reduction of blood glucose compared to injected insulin, but no significant difference was found between these two treatment approaches (Figure 1A, 1B). Serum urea, creatinine, and uric acid in the diabetic group were significantly higher than that in the normal control group (*P*<0.001). Administration of oral insulin-loaded trimethyl chitosan nanoparticles- same as in the case of injected insulin to diabetic rats-obviously attenuated the altered three parameters compared to the untreated diabetic group (*P*<0.05).

***Effect of*** ***insulin-loaded trimethyl chitosan*** ***nanoparticles on oxidative stress markers***

The results of oxidative stress markers are shown in Figure 2 (A to E). As summarized in Figure 2A and 2B, diabetes causes a notable decrease in TAC and increase in TOS level (*P*<0.001). Treatment with oral-coated form and subcutaneous non-coated form of insulin—with no obvious differences between the two—could ameliorate the oxidative markers through a notable increase in TAC and decrease in TOS (*P*<0.05). Furthermore, a significant increase in malondialdehyde (MDA) level was observed in STZ-injected rats versus normal control rats (*P*<0.001). Insulin treatment in both coated and non-coated forms could remarkably attenuate this elevation compared to the untreated diabetic group (*P*<0.05). There was no significant discrepancy in this marker between these two kinds of treatments (Figure 2C). The thiol groups were also markedly reduced in diabetic rats compared to the normal rats (*P*<0.001). Treatment with oral insulin-loaded trimethyl chitosan nanoparticles and injected insulin in diabetic rats showed a significant elevation in thiol groups in comparison with normal group (*P*<0.05) (Figure 2D). As shown in Figure 2E, there was also a significant reduction in catalase activity in the diabetic group versus the normal control group (*P*<0.001). Oral treatment with insulin-loaded trimethyl chitosan nanoparticles, similar to conventional insulin, could considerably compensate the reduced activity of catalase compared to control group (*P*<0.05).

***Effect of*** ***insulin-loaded trimethyl chitosan*** ***nanoparticles on AGE level***

The AGEs level in kidney tissue was significantly higher in diabetic group compared to the normal control group (*P*<0.05). However, the administration of oral insulin-loaded trimethyl chitosan nanoparticles and injected insulin did not show significant differences compared to the normal control group (Figure 3).

***Effect of*** ***insulin-loaded trimethyl chitosan*** ***nanoparticles on RAGE expression***

mRNA expression of RAGE was examined in the kidney tissue. As shown in Figure 4, RAGE mRNA levels are significantly higher in diabetic group compared to the normal control group (*P*<0.001). However, the administration of oral insulin-loaded trimethyl chitosan nanoparticle and injected insulin decreased RAGE expression respectively 14% and 10% compared to the normal control group, but this change was not statistically significant.

## Discussion

DM is the main cause of ESRD, which is associated with an increased risk of morbidity and mortality (4). Hyperglycemia can accelerate DN by AGE-RAGE signaling pathway and oxidative stress activation (1). Inhibition of AGE formation, blockade of the RAGE-signaling pathway, or oxidative stress suppression may widely lead to the amelioration of DN (22). Therefore, the control of blood glucose is a potential therapeutic choice in the prevention of kidney damage. STZ-induced diabetic rats are considered as a proper experimental model for the induction of DN, since the selective damage to pancreatic beta-cells by STZ leads to the poor sensitivity of insulin for glucose uptake (23). Subcutaneous administration of insulin is a conventional method for the management of diabetes mellitus. However, this is an invasive therapy with some disadvantages, such as hypoglycemia, peripheral hyperinsulinemia, uncontrolled drug delivery to the target tissue, poor pharmacodynamics, and risk of infections (9). Recent studies on oral delivery of drugs focus on increased bioavailability and absorption of oral peptides. This route of administration decreases the complications associated with injected insulin. Orally delivered insulin, similar to physiological insulin secretion, is transmitted directly from the intestine to the liver and does not lead to peripheral hyperinsulinemia, providing better glucose homeostasis (24). But, there are some physicochemical barriers in the gastrointestinal tract that hamper the passage of insulin to the bloodstream, such as pH inactivation and enzymatic degradation, intestinal epithelium tight junctions, and short retention time of drugs in absorption site (12). The use of nanocarriers is the best way to prevent the gastrointestinal destruction of drugs and increase permeability in the intestinal epithelium (24). Biocompatibility, biodegradability, high drug loading, and drug protection against degradation are the benefits of nanocarriers for drug delivery (25). Chitosan is a mucosal adhesive polymer that results from the ionic bonding between a positively charged amine group of chitosan and functional groups with a negative charge at the epithelial cell surface. Chitosan is also able to temporarily open tight junctions between epithelial cells and cause protein transmissions (9). Due to the presence of quaternary ammonium the use of trimethyl chitosan, an alkylated derivate of chitosan, can create a constant positive charge in a wide pH range where the polymer remains soluble throughout the digestive system (26). The ionic regulation and polyelectrolyte complexation (PEC) methods are extensively used to prepare the chitosan-alginate nanoparticles (27). According to the aforementioned statement, oral insulin-loaded trimethyl chitosan nanoparticles—in addition to maintaining the hypoglycemic properties of injectable insulin—seems to be more effective in reducing diabetic complications. Therefore, this study examined the effect of oral insulin-loaded trimethyl chitosan nanoparticles on RAGE mRNA expression and oxidative stress status in the renal tissue of STZ-induced diabetic rats. Prusty *et al.* reported that in the group treated with injected insulin during the first hours, the blood glucose levels decrease significantly, which was more than the glucose reduction in the insulin-loaded trimethyl chitosan nanoparticles treated group. Also, over time, blood glucose elevation in this group was more rapid compared to that in the insulin-loaded trimethyl chitosan nanoparticles treated group (28). 

In insulin deficiency, the tissues are unable to uptake glucose to acquire energy, resulting in proteolysis in muscle tissue and lipolysis in adipose tissue, which ultimately leads to weight loss in diabetic animals (29). In this study, treatment with insulin-loaded trimethyl chitosan nanoparticles remarkably increased the body weight more effectively than injected insulin. Oral insulin-loaded trimethyl chitosan nanoparticles were distributed systemically in the body and increased the glucose uptake of the liver, muscle, and adipose tissues and elevated the synthesis of glycogen, fatty acids, and proteins (29). Given that insulin injection keeps insulin levels up for a short time, it has a much lower effect on weight gain than oral insulin-loaded trimethyl chitosan nanoparticles. 

As a result of protein breakdown in diabetic rats, especially in skeletal muscle, serum urea levels are elevated (30). On the other hand, by decreasing the renal filtration in diabetes, this elevation is exacerbated (31). In line with Kuhad and Chopra (2009) our findings showed that administration of the encapsulated form of insulin- similar to that of non-encapsulated form-can reduce serum urea level (32). In fact, insulin decreases the production of urea by reducing protein degradation and also improves renal function, leading to increased excretion of urea.

Uric acid is one of the most important endogenous antioxidants and the final product of purine metabolism. An increase in serum levels of uric acid is due to increased production or reduced excretion (33). Studies show that with increased oxidative stress in diabetes, the level of uric acid increases as an antioxidant defense mechanism (34). This increase is exacerbated by increasing the renal filtration (31). In our study, it is found that treatment with oral insulin-loaded trimethyl chitosan nanoparticles and injectable insulin improves renal excretion of uric acid and reduces uric acid production by improving oxidative stress conditions and decreasing the serum levels of this factor.

Creatinine-as a byproduct of only phosphocreatine decomposition-is excreted by the kidneys. Therefore, measuring the amount of creatinine in the blood can indicate kidney function (34). In this study, increased serum creatinine level in diabetic rats indicated renal injury, but in groups receiving an oral coated form of insulin-same as those receiving injected noncoated form-there was a significant decrease in creatinine levels. In line with this study, Haidara *et al.* reported that insulin could reduce serum urea and creatinine levels in STZ-diabetic rats (35).

Impairment of the oxidant-antioxidant balance results in many pathological conditions such as diabetes and leads to oxidative stress and cellular damage (2). Hyperglycemia leads to mitochondrial superoxide overproduction. This free radical causes the activation of four pathways involved in the diabetic pathogenesis such as a polyol, hexosamine, protein kinase C, and AGE pathway (36). Our study demonstrated a reduction in TAC and elevation of TOS in diabetic rats. Insulin treatment in either form could significantly ameliorate these parameters. This improvement in the oxidative status is due to a diminution of blood glucose. Thus, a reduction in the production of ROS and an improvement in the function of antioxidant systems are observed.

In hyperglycemic conditions, the produced superoxide radicals are converted to hydrogen peroxide by SOD enzyme. H_2_O_2_ is also converted to hydroxyl radical; this radical invades the unsaturated fatty acid chain and forms MDA (37). In the present study, there was a significant increase in the level of MDA in diabetic control groups. Treatment with oral insulin-loaded trimethyl chitosan nanoparticles and injected insulin significantly reduced this factor compared to the diabetic control groups. Along with our results, the findings of Haidara *et al.* revealed that insulin treatment can significantly reduce the increased levels of MDA in the kidney tissue of diabetic rats (35). Thiol compounds-one of the most important antioxidant sources in the cell-are susceptible to oxidative damage and decrease in these conditions (19). In the present study, the levels of thiol compounds in diabetic rats showed a significant decrease compared to those in nondiabetic ones. But treatment with oral insulin-loaded trimethyl chitosan nanoparticles and injected insulin markedly increased this factor compared to diabetic groups. Samadder *et al.* showed that orally delivered insulin-loaded Poly (lactic-co-glycolic acid) (PLGA) nanoparticles-similar to subcutaneous insulin-could elevate the reduced levels of thiol compounds in diabetic rats (29).

Catalase is an important antioxidant enzyme that protects cells against oxidative damage by neutralizing ROSs (20). In our study, there was a significant decrease in the activity of this enzyme in diabetic control groups. However, oral delivery of insulin-loaded chitosan nanoparticles-similar to subcutaneously injected insulin-obviously increased the activity of this enzyme compared to diabetic groups. In consistent with our findings, Kuhad *et al.* also reported that insulin treatment could increase catalase activity in diabetic rats (32).

In hyperglycemic condition, glycation of the amine groups of proteins, lipids, and nucleic acids leads to AGE formation and increase. This reaction occurs over a period of days (38). Both enhanced formation and decreased clearance lead to AGE accumulation in the kidney (4). AGE accumulation on the collagen of the basement membrane and its ability to trap plasma proteins causes a thickening of the basement membrane, change in filtration, and eventual loss of glomerular function (39). Additionally, the AGE-RAGE interaction activates intracellular signaling pathways, which might induce a proinflammatory response, oxidative stress, and apoptosis in the cell (40). Inhibition of AGE formation or blockage of the downstream RAGE pathway can be considered as a therapeutic strategy for DN (41). Studies show that insulin, by activating the PI_3_K signaling pathway, increases the AGE clearance by macrophage scavenger receptors (MSRs) (42). Furthermore, glucose reduction leads to decreased AGE formation. In this study, AGE levels in diabetic control group showed a significant increase compared to healthy control group. Treatment with oral insulin-loaded trimethyl chitosan nanoparticles and injectable insulin-despite the decrease in AGE levels-did not statistically result in remarkable changes. The RAGE mRNA expression in diabetic groups was significantly higher than that of healthy controls. While treatment with oral insulin-loaded trimethyl chitosan nanoparticles and injected insulin reduced the expression of this gene; however, it was not statistically significant.

Regarding the fact that the RAGE expression depends on the duration of exposure to AGE and its concentration (40), also since in this study, treatment with encapsulated insulin and non-encapsulated form did not result in a significant decrease in AGE levels, no significant reduction of RAGE mRNA expression was expected. This unmarked reduction may be due to a short treatment duration or low-dose insulin administration. The amount of RAGE protein in the kidney should also be measured to provide a precise justification for the lack of alteration in expression of the RAGE mRNA.

## Conclusion

The results of this study showed that oral insulin-loaded trimethyl chitosan nanoparticles, along with subcutaneous insulin, can reduce blood glucose and improve renal damage caused by hyperglycemia. In most cases, however, the effect of insulin-loaded trimethyl chitosan nanoparticles on reduction of blood glucose, improvement of oxidative stress conditions, and reduction in AGE levels as well as expression of RAGE was slightly higher than that of injected insulin. Furthermore, insulin-loaded trimethyl chitosan nanoparticle did not have the side effects of injected insulin. Therefore, oral administration of insulin-loaded chitosan/alginate nanoparticles could be a good choice to improve the treatment of type 1 diabetes and should be investigated further in future studies.
